# Alleviation of Osmotic Stress Effects by Exogenous Application of Salicylic or Abscisic Acid on Wheat Seedlings

**DOI:** 10.3390/ijms140713171

**Published:** 2013-06-26

**Authors:** Izabela Marcińska, Ilona Czyczyło-Mysza, Edyta Skrzypek, Maciej T. Grzesiak, Franciszek Janowiak, Maria Filek, Michał Dziurka, Kinga Dziurka, Piotr Waligórski, Katarzyna Juzoń, Katarzyna Cyganek, Stanisław Grzesiak

**Affiliations:** The *F. Górski* Institute of Plant Physiology, Polish Academy of Sciences, Kraków 30-239, Poland; E-Mails: czyczylo@ifr-pan.krakow.pl (I.C.-M.); skrzypek@ifr-pan.krakow.pl (E.S.); m_grzes@ifr-pan.krakow.pl (M.T.G.); fjanowiak@yahoo.com (F.J.); mariafilek@gmail.com (M.F.); michal.dziurka@gmail.com (M.D.); tobola@ifr-pan.krakow.pl (K.D.); pewalig7@gmail.com (P.W.); katarzynajuzon@gmail.com (K.J.); katarzyna_malek@wp.pl (K.C.); grzesiak@ifr-pan.krakow.pl (S.G.)

**Keywords:** abscisic acid, salicylic acid, hydroponic culture, osmotic stress, drought tolerance, yield, wheat

## Abstract

The aim of the study was to assess the role of salicylic acid (SA) and abscisic acid (ABA) in osmotic stress tolerance of wheat seedlings. This was accomplished by determining the impact of the acids applied exogenously on seedlings grown under osmotic stress in hydroponics. The investigation was unique in its comprehensiveness, examining changes under osmotic stress and other conditions, and testing a number of parameters simultaneously. In both drought susceptible (SQ1) and drought resistant (CS) wheat cultivars, significant physiological and biochemical changes were observed upon the addition of SA (0.05 mM) or ABA (0.1 μM) to solutions containing half-strength Hoagland medium and PEG 6000 (−0.75 MPa). The most noticeable result of supplementing SA or ABA to the medium (PEG + SA and PEG + ABA) was a decrease in the length of leaves and roots in both cultivars. While PEG treatment reduced gas exchange parameters, chlorophyll content in CS, and osmotic potential, and conversely, increased lipid peroxidation, soluble carbohydrates in SQ1, proline content in both cultivars and total antioxidants activity in SQ1, PEG + SA or PEG + ABA did not change the values of these parameters. Furthermore, PEG caused a two-fold increase of endogenous ABA content in SQ1 and a four-fold increase in CS. PEG + ABA increased endogenous ABA only in SQ1, whereas PEG + SA caused a greater increase of ABA content in both cultivars compared to PEG. In PEG-treated plants growing until the harvest, a greater decrease of yield components was observed in SQ1 than in CS. PEG + SA, and particularly PEG + ABA, caused a greater increase of these yield parameters in CS compared to SQ1. In conclusion, SA and ABA ameliorate, particularly in the tolerant wheat cultivar, the harmful effects and after effects of osmotic stress induced by PEG in hydroponics through better osmotic adjustment achieved by an increase in proline and carbohydrate content as well as by an increase in antioxidant activity.

## 1. Introduction

Wheat is one of the crops that are exposed to many environmental stresses, limiting their yield. These stresses may be of a biotic or abiotic nature. After being subjected to environmental stresses, plants activate a range of defensive mechanisms which cause, for example, changes in gas exchange [1–4] or accumulation of low molecular osmoprotectants, like carbohydrates, proline or polyamines [5–7]. These mechanisms may also be induced or enhanced by the application of chemicals to the plants.

The application of salicylic acid (SA) has been reported to induce tolerance to osmotic stress [8,9]. The protective effect of SA pretreatment was confirmed by the determination of, among other parameters, malonyldialdehyde (MDA) content. SA pretreatment initially caused formation of reactive oxygen species (ROS) and decreased net photosynthetic rate and stomatal conductance, but prevented ROS accumulation during subsequent osmotic stress by activating antioxidant enzymes. SA, especially at 0.5 mM, alleviated the adverse effects of osmotic stress on most recorded parameters. It is a water-soluble antioxidant compound which can also regulate plant growth [2,10]. SA at lower concentration had a stimulatory effect in wheat plants on photosynthetic electron transport activity and phosphate uptake [11,12]. Exogenous SA also has several physiological and biochemical effects. It may decrease transpiration [2], and inhibit cis-abscisic acid (ABA)-induced stomatal closure. Ameliorative effects of salicylic acid on the growth of crop plants under abiotic stress conditions may be due to its role in nutrient uptake [13]. In the studies of Sakhabutdinova *et al*. [14], high ABA levels were maintained in SA treated wheat seedlings, which induced anti-stress reactions, such as the maintenance of proline accumulation. Sahu and Sabat [15] reported a negative effect of SA on growth and activity of major enzymatic antioxidants.

ABA plays a special role in the resistance of plants to water deficit or osmotic stress caused by soil drought. Usually the level of ABA increases in plants during stress conditions and is associated with plant defense reactions [16,17]. The main role of ABA in the adaptation and/or resistance of plants to water deficit conditions is the participation in stomatal closure, which reduces water loss through transpiration [18,19]. Under drought stress conditions, ABA also participates in the communication between the root and above-ground part, which needs to quickly adapt to water deficit conditions, e.g., by partial stomatal closure and metabolic changes [20,21]. If there is no water in the soil available to the roots, they produce increased amounts of ABA, which is transported through xylem sap to the shoots, where it causes stomatal closure and reduced transpiration and loss of leaf turgor [22]. ABA is thus used by the plant as an early signal which can be observed before the water deficit in the soil causes significant changes in water relations of the shoots [21].

It was widely accepted that ABA is responsible for abiotic stress signaling, whereas SA (with jasmonic acid and ethylene) takes part in biotic stress reactions. There is much evidence that this simple division is wrong. Different authors provided premises of cross-talk between ABA and SA [23,24] under the stress conditions. The number of publications focused on this problem is increasing, but details of this cross-talk are not clear.

Osmotic stress in plant cells leads to a reduction in carbon assimilation, which is linked to a physiological closure of leaf stomata and to biochemically determined lower photosynthetic activity, which affects carbohydrate economy [25]. Soluble sugars are acting as osmolytes maintaining cell turgor of leaves, protecting the integrity of the membrane, and preventing the denaturation of proteins [7]. Sucrose plays an important role in plant metabolism at both cellular and whole organism level. It participates not only in the response to abiotic stresses, but also serves as a nutrient and signaling molecule, modulating a wide range of gene activity [26].

The class of small molecules known as “compatible osmolytes” also includes proline. Proline accumulation plays a highly protective role in plants that are exposed to abiotic stresses, being involved in osmotic adjustment together with an increase in the levels of other osmolytes [27,28]. Proline has also been suggested to act as antioxidant and to interact with hydrophobic residues in proteins [29,30]. Moreover, at high concentrations, it has little or no perturbing effect on macromolecule-solvent interactions. It was also shown that proline did not impact cell membrane functioning or enzyme activity and contributed to mitigating the negative impact of dehydration [31]. Like ABA, proline is associated with adaptation to stress, causing the expression of genes which protect cells against dehydration.

Polyamines (PAs), mainly diamine putrescine (Put), triamine spermidine (Spd), and tetraamine spermine (Spm), are polycationic compounds of low molecular weight which are present in all living organisms. They have been proposed as a new category of plant growth regulators that can be involved in a large spectrum of physiological processes, and in addition, they have been shown to be an integral part of plant stress response [6,15,32]. Liu *et al*. [11] reported that when two wheat cultivars with different degrees of drought tolerance were treated with PEG, a marked increase in free Spd and Spm was observed in the tolerant cultivar, while a significant increase in free Put was detected in the sensitive cultivar.

The aim of the present work was to evaluate the effect of osmotic stress caused by PEG in hydroponic culture on wheat seedlings and to assess the role of SA or ABA application by studying seedling morphology as well as biochemical and physiological changes. An additional aim was to determine the putative signalling/osmoprotectant role of PAs under stress conditions and their reaction to the expected alleviating effect achieved by the application of SA or ABA during the stress.

## 2. Results and Discussion

It was shown that the optimal level of osmotic stress—defined as most suitable for differentiating drought resistance of SQ1 and CS wheat cultivars based on the differences in the response to osmotic stress caused by PEG—was −0.75 MPa [33]. Osmotic stress was found to affect water status, seedlings morphology, gas exchange parameters, chlorophyll content, osmotic potential, lipid peroxidation, water soluble sugars and proline contents in drought resistant and drought susceptible wheat cultivars. In this study we evaluated the influence of PEG solution in hydroponics culture supplemented with ABA and SA on osmotic stress amelioration in CS and SQ1 cultivars.

### 2.1. Physiological and Morphological Seedling Characteristics under Osmotic Stress

The concentration of 0.05 mM SA or 0.1 μM ABA in solution containing half-strength Hoagland medium (control, C) and PEG 6000 caused a number of physiological changes in the examined wheat cultivars, among others: in water relations, gas exchange, chlorophyll content, morphological traits and MDA content.

#### 2.1.1. Water Relations

**Figure 1:**
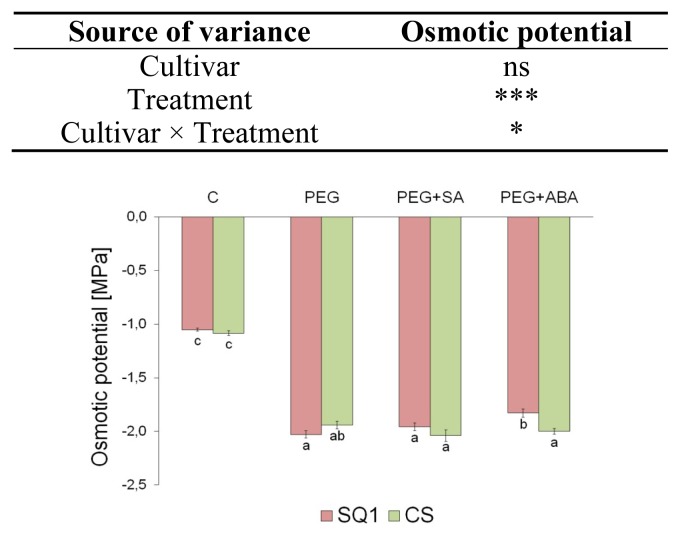
Osmotic potential in leaves of two wheat cultivars: CS and SQ1 after seven days of treatment in half-strength Hoagland solution (control, C) and supplemented with PEG, PEG with SA (PEG + SA) and PEG with ABA (PEG + ABA). Each bar represents a mean of 3 replicates ± SE. Values marked with the same letter do not differ significantly at *p* ≤ 0.05 according to Duncan’s multiple range test. The results of two way ANOVA analysis of variance are presented in the table above the figure. The sources of variance for osmotic potential were as follows: two cultivars, four treatments, and interaction between cultivar and treatment. *, **, ***, significant at *p* ≤ 0.05, 0.01, 0.001, respectively; ns, not significant.

Water content did not significantly differ between cultivars but did differ slightly between treatments, which was confirmed by two-way ANOVA analysis of variance (Table 1). No significant differences were observed in osmotic potential of the cultivars, either. After PEG treatment, osmotic potential decreased by about 98% in comparison to control (Figure 1). Supplementing the medium with SA did not change the value of this parameter, whereas supplementing it with ABA increased it by about −0.2 MPa only for susceptible SQ1 cultivar. Decrease of turgor in plant tissues grown under stress conditions is a typical plant reaction to different abiotic stresses. Decrease of water content and osmotic potential is caused by the injury of cell membrane. According to Nemeth *et al*. [34], SA caused an increase in electrolyte leakage in maize and wheat. A similar effect was observed by Luo *et al*. [1] in barley. ABA improves water relations under water, temperature and salt stress [35]. Application of exogenous ABA under water stress improved the maintenance of leaf water potential and relative water content by stomata closure and increased root water uptake, and, additionally, improved membrane stability by reducing electrolyte leakage [36].

#### 2.1.2. Gas Exchange and Chlorophyll Content

After PEG treatment net photosynthesis, transpiration, water use efficiency, stomatal conductance, and chlorophyll content decreased more for resistant CS than for susceptible SQ1. The addition of SA or ABA to the solution did not change the values of these parameters. ANOVA analysis of gas exchange parameters and chlorophyll content showed significant differences between CS and SQ1 for all parameters, both under control and three treatments (PEG, PEG + SA and PEG + ABA) (Table 2). Variation amongst treatments was significant for Pn and WUE. After PEG treatment lasting 7 days, net photosynthesis (Pn), transpiration (E), water use efficiency (WUE), stomatal conductance (g^s^) and chlorophyll content (SPAD) significantly differed between cultivars and decreased more for resistant CS than for susceptible SQ1. Pn for CS decreased by 51% and for SQ1 only by 27% in comparison to control. Decrease of E was smaller at 28% and 11%, respectively. Comparable decreases in the values of WUE and g^s^ were observed: for WUE: 29 and 12%, and for g^s^: 54 and 44%, respectively. The differences between CS and SQ1 for treatments PEG+SA and PEG+ABA were not high. Chlorophyll content (SPAD) decrease for CS was slightly greater (31%) than for SQ1 (27%). The results of gas exchange parameters (Pn, E, g^s^, WUE) obtained in this study indicate typical plant response to osmotic stress According to the findings of Grzesiak *et al*. [37], under drought conditions carbon dioxide assimilation and transpiration rate decreased and stomatal conductance increased. In the present study, osmotic stress (−0.75 MPa) decreased the values of Pn, E, g^s^, WUE and SPAD to a greater extent in drought resistant CS in comparison to drought sensitive SQ1. Exogenous application of salicylic acid (PEG + SA) or abscisic acid (PEG + ABA) did not cause any significant changes in the measured traits in comparison to PEG treatment. Several reports support the view that SA and ABA may have a protective function against drought and other abiotic stresses. Luo *et al*. [1] suggested an immediate protective effect of SA during osmotic stress on photosynthetic rate and stomata conductance in barley. In a study by Nemeth *et al*. [34], the use of SA with PEG caused a decrease in gas exchange parameters in maize and wheat and changes in chilling and drought tolerance. Similarly, in a study by Janda *et al*. [38], SA pre-treatment decreased net photosynthesis, stomatal conductivity, transpiration and chilling injures in maize. According to Najafian *et al*. [2], the application of SA increased photosynthetic rate and water use efficiency in salt stressed thyme plants. Transpiration rates and stomatal conductance were significantly lower in SA treated plants [18,19]. Beneficial effects of SA in saline conditions such as maintaining gas exchange parameters may have contributed to the reduction of total surface of leaf necrosis. Similarly, Yordanova and Popova [4] suggested that the exposure of wheat to a low temperature decreased chlorophyll content, CO2 assimilation and transpiration rate. Application of SA resulted in a lower photosynthesis rate, decreased transpiration and stomatal conductance, accompanied by enhanced lipid peroxidation rate and peroxide level. ABA reduced water loss through transpiration and significantly decreased stomatal conductance in *Picea asperata* [39].

#### 2.1.3. Morphological Traits and MDA

After seven days in the presence of PEG in the medium, leaves and root length was reduced by about 24% and 38%, respectively, in seedlings of susceptible SQ1 cultivar, and remained at the same level in resistant CS in comparison to C (Table 1). This data is in agreement with the results obtained in our previous experiment [33]. Supplementing SA or ABA to the medium (PEG + SA or PEG + ABA) caused a decrease of these seedling parameters for both cultivars. Similar observations were presented in a study by Sahu and Sabat [15], where SA was added to the culture of wheat seedlings in the early stage of growth under salinity stress conditions. ABA application significantly decreased dry shoot biomass and significantly increased leaf mass per area, root/shoot ratio, and fine root/total root ratio in *Picea asperata* [39]. Sakhabutdinova *et al*. [14] reported that the influence of PEG on wheat seedlings led to a noticeable and almost equal extent of inhibition of growth of plants both treated and not treated with SA. The presented data thus indicate that pretreatment of seeds with SA contributes to the increase in the resistance of plants to environmental stress factors through the manifestation of the protective role of SA. SA treatment induces high accumulation of endogenous ABA, an inducer of a wide spectrum of anti-stress reactions in plants, which is the reason why the influence of SA on the increase of ABA probably lies at the root of the preadaptive role of SA in countering stress conditions. Maintaining a high level of ABA in SA-treated plants under stress contributes to protective reactions aimed at decreasing its injurious effect on growth and acceleration of growth resumption. The osmotic shock caused by PEG treatment resulted in a dramatic inhibition of growth in tomato [9]. Hussain *et al*. [8] demonstrates that SA application after exposure to salinity stress increases survival and decreases the severity of stress injuries in thyme seedlings. In the presence of SA, leaves accumulated different compatible osmolytes, such as sugars, sugar alcohol and proline. In the case of PEG-induced osmotic stress, the acclimation of plants pre-treated with SA successfully occurred under the conditions of highly reduced antioxidative enzyme activity at the initial stage of the stress [2,10].

**Figure 2:**
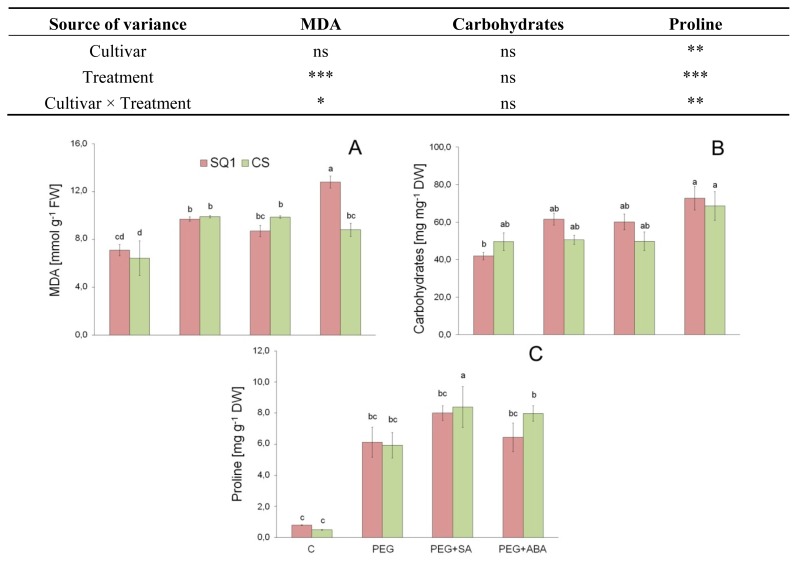
MDA, soluble carbohydrates and proline content in leaves of two wheat cultivars: CS and SQ1 after seven days of treatment in half-strength Hoagland solution (control, C) and supplemented with PEG, PEG with SA (PEG + SA) and PEG with ABA (PEG + ABA). Each bar represents a mean of 3 replicates ± SE. Values marked with the same letter do not differ significantly at *p* ≤ 0.05 according to Duncan’s multiple range test. The results of two way ANOVA analysis of variance are presented in the table above the figure. The sources of variance for MDA, carbohydrates and proline were as follows: two cultivars, four treatments, and interaction between cultivar and treatment. *, **, ***, significant at *p* ≤ 0.05, 0.01, 0.001, respectively; ns, not significant.

Lipid peroxidation, measured as MDA content in leaves, did not differ between cultivars, but did differ between treatments. It was shown that MDA content increased by 20% after PEG and PEG + SA treatment for both cultivars (Figure 2A). Lower MDA content means higher antioxidative ability, reflecting higher drought resistance, as suggested by Shao *et al*. [24]. PEG + ABA treatment caused a further increase of this parameter for susceptible SQ1 while it remained on the same level for resistant CS. Similarly, in investigations by Sahu and Sabat [15], it was noticed that exogenously applied SA increased MDA content in wheat plants, though only when applied in higher concentration. They concluded that SA in low concentration can regulate the activities of intracellular antioxidant enzymes and increase plant tolerance to environmental stresses. Luo *et al*. [1] confirmed that application of exogenous SA in low concentration markedly decreased MDA content of leaves, suggesting that SA enhanced osmotic stress tolerance of barley. In contrast, when SA was applied in higher concentration, MDA content increased, indicating that SA aggravated osmotic stress injury and itself could be another type of stress. Relative to control plants, cold-treated wheat plants showed increased levels of MDA [4]. Thus, these results confirmed the well-known effect of cold stress on membrane integrity. ABA application significantly decreased MDA content improving membrane integrity under water-deficit conditions in a dry climate population [36].

### 2.2. Osmoprotectants, ABA and Antioxidants in Seedling Leaves under Osmotic Stress

#### 2.2.1. Carbohydrates

Concentration of soluble carbohydrates in leaves of both cultivars was comparable for all treatments, though it was visibly higher for the seedlings grown on media modified by PEG, SA and ABA in comparison to control (Figure 2B). The highest values were observed in the presence of ABA. Similarly, Khan *et al*. [40] noted that carbohydrate content significantly increased after exogenously supplementing ABA in soaking seeds of wheat. Soluble sugars and proline are major constituents of osmoregulation in leaves [41]. The accumulation of sugars in response to drought stress is well documented [42,43]. A complex essential role of soluble sugars in plant metabolism is well known; they are products of hydrolytic processes—substrates in biosynthesis processes and energy production—but also play a role in sugar sensing and signaling systems. It has been recently claimed that under drought stress conditions, sugar flux may even be a signal for metabolic regulation [44]. Soluble sugars may also function as typical osmoprotectants, stabilizing cellular membranes and maintaining turgor pressure. According to Khan *et al*. [40], SA and ABA in wheat seeds treatment could be treated as a potential inducer of drought tolerance in wheat.

#### 2.2.2. Proline

Two-way ANOVA analysis of variance confirmed the significant differences in the changes of proline concentration in leaves dependent on the cultivar and treatment (Figure 2C). The interaction between cultivar and treatment was also significantly different. After PEG treatment, proline content increased about six-fold in comparison to control for both cultivars. Supplementation of medium with SA and ABA increased proline content only for resistant CS, though more in the case of SA. It is well known that proline accumulates in plants during adaptation to various types of environmental stresses (for example drought). Different roles have been proposed for proline accumulation and the presented data supports the notion that proline accumulation in stressed plants has a protective function. It is suggested that the glutamate pathway, rather than the ornithine pathway, plays a vital role in proline accumulation in plants exposed to environmental stresses [45]. The accumulation of proline is also dependent on its degradation catalyzed by mitochondrial proline dehydrogenaze (PDH) in plant cells [44], though it contributes little to proline accumulation in wheat [46]. In addition to induction or activation of enzymes of proline biosynthesis or decreased proline oxidation to glutamate, proline accumulation under stress conditions may be caused by decreased utilization of proline in protein synthesis and enhanced protein turnover [45]. Proline metabolism is unique in that polyamines share common substrates, ornithine and glutamate, through their mutual conversion to glutamate-gamma-semialdehyde, whereas these metabolites often respond to abiotic stresses. Thus proline accumulation could be a cause of decreased polyamine content in stressed plants. It is possible, though, that a common signal (possibly ABA) triggers all the sub pathways in a coordinated manner.

#### 2.2.3. Polyamines

**Figure 3:**
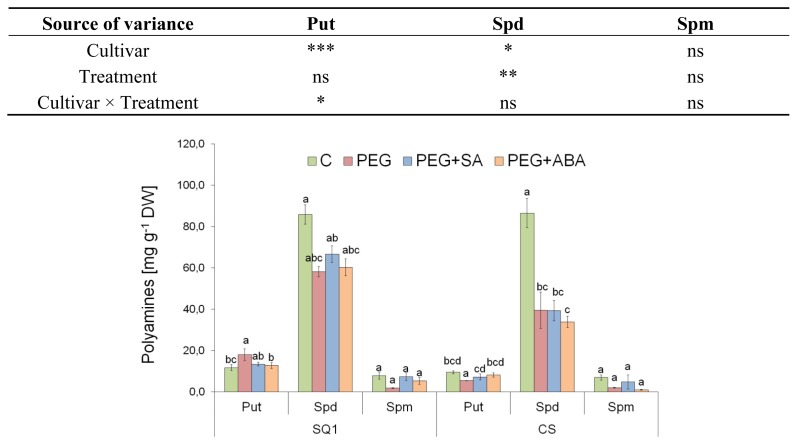
Polyamines (putrescine-Put, spermidine-Spd, and Spermine-Spm) content (μg/mg^−1^ DW) in leaves of two wheat cultivars: CS and SQ1 after five days of treatment in half-strength Hoagland solution (control, C) and supplemented with PEG, PEG with SA (PEG + SA) and PEG with ABA (PEG + ABA). Each bar represents a mean of 3 replicates ± SE. Values marked with the same letter do not differ significantly at *p* ≤ 0.05 according to Duncan’s multiple range test. The results of two way ANOVA analysis of variance are presented in the table above the figure. The source of variance for Put, Spd and Spm were as follows: two cultivars, four treatments, and interaction between cultivar and treatment. *, **, ***, significant at *p* ≤ 0.05, 0.01, 0.001, respectively; ns, not significant.

Endogenous accumulation of Put and Spd in seedling leaves differed significantly between cultivars, though only for Spd between treatments (Figure 3). The lowest accumulation was observed for the third polyamine, Spm, and was statistically different neither for cultivar, nor for treatment. PEG treatment caused an increase of Put content by about 35% only in susceptible SQ1 in comparison to control. Spd content was several times higher than other polyamine content in both cultivars. Biosynthesis of Spd under PEG treatment decreased for both cultivars compared to control by about 32% and 54% for CS and SQ1, respectively. Only SA addition to the medium slightly increased Spd content for susceptible SQ1. It is a widely accepted hypothesis that polyamines can act as osmoprotectants [32]. This is because of their chemical property as a polycation, which allows them to bind high amounts of water and to stabilize the structure of the membranes. Many authors report a significant increase of endogenous polyamines level under abiotic stresses [32], though there are some papers describing different reactions. Maiale [47] applied salinity stress to two rice cultivars and they found free Put and Spd levels decreased under stress conditions. The observed changes in polyamines content seem to be caused by lowered synthesis, which was showed by Maiale [47] by measuring the activity of some enzymes involved in polyamines anabolism: arginine decarboxylase, *S*-adenosyl-L-methionine decarboxylase, spermidine synthase and the enzyme responsible for their catabolism–polyamine oxidase. They observed that the activity of the enzymes of polyamines biosynthesis was lowered, while polyamine oxidase was not active. This explanation can also be supported by increased ABA level after the treatment in our experiment. ABA is known to induce expression of polyamine biosynthesis genes, and ABA-Responsible Elements (ABRE) [48] are present in the promoter region of the majority of these genes. Another way of decreasing polyamines level can be blocked biosynthesis from any precursor, like arginine or proline. Proline accumulation observed in this study would seem to indicate this phenomenon. A complementary explanation of the decrease in polyamine content under drought stress could be their elevated oxidation, which would be consistent with an increased level of MDA, however more experiments are needed to confirm this hypothesis. Other factors responsible for different results obtained among the researchers can be the duration of the stress. Legocka [49] showed a quick increase of polyamines level in the roots of lupinus under salt stress lasting 4 hours, but unexpectedly, polyamine levels decreased after 24 h of stress. Similar results obtained by Tonon *et al*. [50], indicate that polyamines play their signaling or osmoprotecting roles mainly at the beginning of the stress. As mentioned above, some authors postulated that PAs could compete for the same substrates as proline: arginine and ornitine, but Do *et al*. [51] proved that levels of ornithine and arginine does not strongly influence the level of proline. One possible explanation is that the glutamate pathway is more important for proline biosynthesis than the ornithine pathway. Of course, this does not exclude the impact of increased proline synthesis, as found in our experiments, on lowered concentration of PAs (synthesized from arginine), but it is not the most probable reason.

#### 2.2.4. ABA

**Figure 4:**
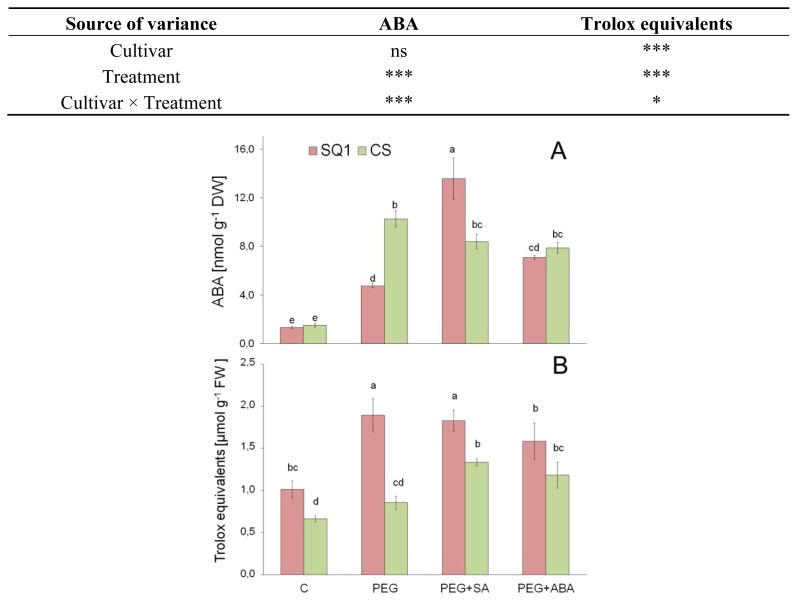
Abscisic acid (ABA) content (nmol g^−1^ DW) and total activity of low molecular antioxidants (trolox equivalents) in leaves of two wheat cultivars: CS and SQ1 after seven days of treatment in half-strength Hoagland solution (control, C) and supplemented with PEG, PEG with SA (PEG + SA) and PEG with ABA (PEG + ABA). Each bar represents a mean of 6 replicates ± SE; two measurements of three samples each collected form three plants per treatment. Values marked with the same letter do not differ significantly at *p* ≤ 0.10 according to Duncan’s multiple range test. The results of two way ANOVA analysis of variance are presented in the table above the figure. The sources of variance for ABA and Trolox equivalents were as follows: two cultivars, four treatments, and interaction between cultivar and treatment. *, **, ***, significant at *p* ≤ 0.05, 0.01, 0.001, respectively; ns, not significant.

ABA level in the leaves of control plants ranged between 1.3 and 1.5 nmol g^−1^ DW and did not differ between the cultivars (Figure 4A). After seven days of PEG treatment, leaf ABA content increased four-fold in susceptible cultivar SQ1 and seven-fold in resistant CS in comparison to control. PEG + SA caused a further increase in ABA content for SQ1 (of about 60%) and a slight decrease for CS. Interestingly, PEG + ABA did not increase ABA level more than other PEG treatments, and moreover, it significantly decreased ABA level in SQ1 compared to PEG + SA. ABA level in wheat leaves observed in the course of this study corresponds to that observed by Quarrie [52], and similar increases of ABA level were observed in Arabidopsis thaliana under osmotic stress [53] as well as in wheat leaves under both osmotic [54] and soil drought stress [55]. ABA increase under stress is a common reaction in the plant kingdom. A greater accumulation of ABA under stress conditions in the resistant cultivar compared to the susceptible one was also observed under other types of stress, e.g., under frost in wheat [56] and under chilling in maize [57]. The higher ABA level in the tolerant cultivar increases its stress tolerance through stomata closure reducing transpiration and improving water relations (Table 1, Figure 1), and alleviates the negative after effects of stress on the yield (Table 3). The long-term ameliorating effect of ABA under osmotic stress is achieved through ABA-mediated transcriptional regulation of a number of genes [58]. The direct cause of leaf ABA increase under osmotic stress induced by PEG in hydroponics does not seem to be the dehydration of leaf tissue because leaf water content dropped only by 5% (Table 2). Most probably the reason for increased leaf ABA was the dramatic drop in osmotic potential of leaves under PEG treatment (Figure 1). Though the addition of ABA at the concentration of 0.1 μM to nutrient solution containing PEG did not increase endogenous leaf ABA level compared to PEG treatment (Figure 4A), it increased proline and carbohydrate level, especially in the resistant cultivar (Figure 2B,C), thereby making an essential contribution to its stress tolerance.

#### 2.2.5. Antioxidants

Total antioxidant activity (TAA) of leaf tissue, as measured by DPPH method, represents the potential antioxidant activity of all non-enzymatic, low-molecular antioxidants of leaf tissue, such as glutathione, ascorbic acid, phenols, polyphenols, flavonoids, anthocyanins, tannins, polysaccharides, tocopherol, proline, betaine, and others [59]. The non-enzymatic antioxidants protect plant cells from oxidative damage by scavenging reactive oxygen species (ROS), play the role of enzyme cofactors, and additionally, are important redox signaling components [60]. TAA under control conditions was significantly higher in SQ1 (1.01 μmoles Trolox equivalents g^−1^ FW) as compared to CS (0.66; Figure 4B). After seven days of PEG treatment TAA increased two-fold in susceptible cultivar SQ1 and only slightly in resistant CS in comparison to control. During PEG + SA and PEG + ABA, TAA levels were still considerably higher in both cultivars as compared to control. However, this level was similar or slightly lower than that of PEG treatment in SQ1, whereas in CS it increased substantially in comparison to PEG treatment. Thus, the addition of SA or ABA to nutrient solution has a genotypically differentiating effect on TAA as compared to PEG treatment—almost no change or a decrease in the susceptible cultivar—and an increase in the resistant one. It may be assumed that the two-fold increase of TAA in susceptible SQ1 under osmotic stress (PEG treatment, −0.75 MPa) is a reaction by wheat seedlings to oxidative stress, mainly caused by higher generation of ROS. The increase of TAA and thus the level of oxidative stress in the resistant cultivar under PEG treatment was significantly smaller (Figure 4B). A similar increase in antioxidant pools in maize seedlings under short-term osmotic stress was observed by Kolarovic *et al*. [61]. In turn, Kellos *et al*. [62] found that not only osmotic stress but also treatment with SA or hydrogen peroxide increased the level of low molecular antioxidants, especially in the stress tolerant maize genotype. In the latter study, ABA caused a smaller increase of antioxidant level in comparison to SA, which is the case in our results as well (Figure 4B). Probably the uptake of exogenous SA by plant roots is more efficient than that of ABA and, additionally, SA acts as an antioxidant in the plant. Under water shortage condition, antioxidant activity in plants usually increases and this adaptational reaction is mediated by ABA [63]. It has also been reported in the literature that antioxidant content was decreased greatly in drought-sensitive cultivars after drought stress and in chilling-sensitive cultivars after chilling stress in contrast to resistant cultivars, where an increase was observed [64].

### 2.3. Aftereffects of Osmotic Stress on Yield Components

It was determined that yield components decreased for CS and SQ1 plants harvested after achieving full maturity in the soil after replacement from hydroponics culture in modified media (PEG, PEG + SA and PEG + ABA) (Table 3). ANOVA analysis showed significant differences dependent on the cultivar for all yield components and on the treatment for grain mass and biomass. The decrease of these parameters after PEG treatment was higher by 10% for susceptible SQ1 then for CS. Supplementation of the solution with SA slightly increased yield components in both cultivars. Supplementation with ABA increased the values of these parameters more than SA, though only for resistant CS, while a decrease was observed for SQ1, even up to 50%, in comparison to control. Growth and yield of wheat have been seriously influenced by drought in many regions. Khan *et al*. [40] demonstrated that treatment of wheat seeds with SA and ABA was reflected in increased grain yield in the genotypes investigated. This suggests that the vegetative stage may be critical for the growth, subsequent development and yield of wheat under drought conditions, which was confirmed by Loutfly *et al*. [65]. It was observed that economically profitable yields of wheat genotypes grown under water stress could be maintained if their seedlings were treated with SA or ABA. Loutfly *et al*. [65] showed that the growth rate might be determined by water sensitive processes other than photosynthesis. In wheat, some proteins and carbohydrates increased under drought stress, although total biomass decreased. Their levels could be maintained by primary metabolism including photosynthetic assimilation and normal transport activities. Cairns *et al.* [66] showed that grain yield and drought adaptation in maize are complex traits, which make breeding gains slow. Yield is a function of many processes throughout the plant cycle and instantaneous measurements only provide a snapshot of a given plant process. Drought stress significantly reduced yield and yield components. Yield loss in maize was largely associated with a highly significant decrease in the number of kernels per cob.

## 3. Experimental Section

### 3.1. Plant Material

Seedlings of drought resistant (Chinese Spring, CS) and drought susceptible (SQ1) hexaploid wheat (*Triticum aestivum* L.) were examined in the experiment. The genotype SQ1 was selected at the Plant Breeding Institute, UK, from the 7th cross generation (F^7^) between two wheat cultivars: Highbury × TW269/9/3/4. CS and SQ1 differ significantly in their physiological, morphological, and developmental traits. In comparison to CS, SQ1 is shorter with a smaller leaf surface area, and fewer spikes which have awns [52].

### 3.2. Experimental Design

Grains were surface-sterilised with 96% ethanol for 1.5 min followed by 8 min in 5% calcium hypochlorite [Ca(ClO)^2^], before being washed four times in sterile water. Afterwards, grains were germinated on wet filter paper for 3 days. Germinating seedlings were put into plastic containers filled with half-strength Hoagland solution and maintained in hydroponics culture in a greenhouse for 21 days. Seedlings were grown at 25 °C under a 16 h photoperiod and 400 μmol m^−2^ s^−1^ light intensity. The hydroponic solution was aerated by air pumps. Every day the hydroponic medium was supplemented with fresh medium and every week it was completely exchanged with fresh medium. The seedlings of each cultivar were grown until the fourth leaf was fully expanded. Then they were randomly divided into four groups: C–control seedlings, grown only in half-strength Hoagland solution, PEG–grown in C + PEG 6000 (−0.75 MPa) causing osmotic stress (PEG), PEG + SA – PEG + 0.05 mmol dm^−3^ of salicylic acid, and PEG + ABA–PEG + 0.1 μmol dm^−3^ of abscisic acid. The pH of all solutions was adjusted to 6.0. The seedlings were maintained in these media for the next 7 days. After the collection of the samples, seedlings of all groups, in three replicates, were replanted into soil and grown to maturity. After the harvest, components of yield were determined in order to study the influence of the treatments at the seedling stage on grain number and mass per plant and biomass values.

### 3.3. Measurements and Analysis

As in a previous experiment [33], gas exchange parameters (Pn, E, g^s^) were measured using a CIRAS 2 analyzer (PP System, Hitchin, UK), chlorophyll content was measured with a SPAD CL 01 meter (Hansatech, Norfolk, UK), and leaf osmotic potential with a psychrometer HR 33T (WESCOR). Measurements were performed on the 7th day after applying osmotic stress in 16 replications. On the last (7th) day of osmotic stress, length of leaves, roots, fresh weight (FW) and dry weight (DW) of leaves and roots were measured in order to determine water content [(FW − DW/DW) × 100)]. Seedling samples were collected in three replicates after 7 days of stress for the determination of leaf osmotic potential by Wescor, level of lipid peroxidation (concentration of malondialdehyde, MDA) as in Dhindsa *et al*. [67], contents of soluble carbohydrates as in Dubois *et al*. [68], proline as in Ting and Rouseff [69] with modifications, ABA as in Walker-Simons and Abrams [70], polyamines as in Smith and Davies [71], and total activity of low molecular antioxidants as in Brand-Williams *et al*. [72].

### 3.4. Osmotic Potential

The measurements were performed using a microvoltmeter (model HR-33T with C-52 sample chambers, Wescor Inc., Logan, UT, USA) in the mode of “dew point”. Leaf discs (ø = 5 mm) were collected for analysis from the middle part of leaves and were placed in an Eppendorf tube, frozen in liquid nitrogen and stored at −70 °C. During the measurement, leaf samples were thawed at room temperature and the sap from leaf discs was extracted with a syringe and quickly transferred to a leaf chamber. The time needed for the saturation of leaf chambers was set to 40 min. The measurements for each genotype were taken in the dew point mode at room temperature in 5 replicates.

### 3.5. Lipid Peroxidation (MDA Content)

1 g of fresh leaves was ground in 5 mL of 0.5% trichloroacetic acid (TCA) and centrifuged at 1000× *g* for 15 min. The mixture containing 1 mL of the supernatant and 4 mL of 0.5% thiobarbituric acid (TBA) in 20% TCA was heated at 100 °C for 30 min and then cooled to room temperature. The specific absorbance (at 532 nm) of the extract (relative to the background absorbance at 600 nm) was determined. The concentration of malondialdehyd (MDA) was expressed in μmol g^−1^ FW (fresh weight of leaves), using a molar extinction coefficient equal to 155 × 105 mmol^−1^ cm^−1^.

### 3.6. Soluble Carbohydrates

About 5 mg of lyophilized and homogenized samples were extracted in 1.5 mL of 96% ethanol for 15 min. Then the samples were centrifuged at 21,000× *g* for 15 min and 40 μL of the supernatant was transferred to 10 mL test tubes containing 400 μL of deionised water. Afterwards 400 μL of 5% phenol and 2 mL of concentrated sulphuric acid were added. Samples were incubated for 20 min and transferred to 96-well plates. Absorbance was measured at 490 nm on a micro-plate reader (Synergy 2, Bio-Tek, Winooski, VT, USA). The level of carbohydrates was expressed in μg g^−1^ DW (dry weight of leaves).

### 3.7. Proline

About 5 mg of lyophilized and homogenized samples were extracted in 0.5 mL of 3% 5-sulphosalicylic acid for 15 min. Then the samples were centrifuged at 21,000× *g* for 15 min. The clear supernatant (200 μL) was transferred to polypropylene screw cap vials, after which 200 μL of concentrated formic acid and 400 μL of 3% ninhydrin reagent in 2-methoxyethanol were added. Samples were heated for 0.5 h at 100 °C in a water-bath, and then transferred to 96-well plates. Absorbance was measured at 514 nm on a micro-plate reader (Synergy 2, Bio-Tek, Winooski, VT, USA). The level of proline was expressed in μg g^−1^ DW (dry weight of leaves).

### 3.8. Abscisic Acid

Plant material was freeze-dried and samples were ground with ball mill MM400 (Retsch, Haan, Germany) in Eppendorf vials, to which 1.5 mL of cold distilled water was then added. The vials were then placed in boiling water for 3 min and shaken overnight at 4 °C. The next day, the extracts were centrifuged for 20 min in a refrigerated centrifuge at 18,000× *g* (MPW-350R, Warsaw, Poland). ABA was measured in the supernatant using indirect enzyme-linked immunosorbent assay (ELISA). The antibody used was MAC 252 (Babraham Technix, Cambridge, UK). Absorbance was measured by microplate reader Model 680 (Bio-Rad Laboratories, Inc., Hercules, CA, USA) at the wavelength of 405 nm. For each treatment, at least six independent ELISA measurements were made on three independent samples collected from three different plants.

### 3.9. Total Activity of Low Molecular Antioxidants (TAA)

Plant material was ground with ball mill MM400 (Retsch, Germany) in Eppendorf vials, to which 50% ethanol was then added and shaken for two hours at room temperature. The extracts were then centrifuged for 20 min in a refrigerated centrifuge at 18,000× *g* (MPW–350R, Warsaw, Poland) and the supernatant was used for the measurements. The total content of antioxidants (free radical-scavenging activity) in the tissues was measured by DPPH method according to Brand-Williams *et al*. [72] with some modifications adapting the protocol to 96-well microtitre plates and to the measurement of absorbance by microtitre plate reader [73]. A solution of 0.5 mM of stable free radical 1,1-diphenyl-2-picrylhydrazyl (DPPH, SIGMA) in methanol was used. Absorbance was determined after 30 min of the reaction at 37 °C at 490 nm using reader Model 680 (Bio-Rad Laboratories, Hercules, CA, USA). The results were expressed as μmoles of Trolox equivalents. For each treatment, at least six independent measurements were made on three independent samples collected from three different plants.

### 3.10. Polyamines

Lyophilized samples were homogenized in ball mill MM400 (Retsch, Germany). A 0.02 g portion of the sample was extracted in 1 mL 5% HClO^4^ and sonicated for 10 min. Afterwards the samples were centrifuged under 37,000× *g* for 10 min and the supernatant was collected. Extraction was repeated and supernatants were combined. 200 μL of combined supernatant were transferred to 2 mL polypropylene tubes and neutralized with 10 μL saturated NaOH, after which 400 μL dansyl chloride solution (5 mg/mL in acetone) and 200 μL saturated sodium carbonate solution were added. Samples were incubated at room temperature overnight. Afterwards, proline solution (100 mg/mL in water) was added and the mixture was incubated for 30 min. Finally, dansylated polyamines were extracted to 750 μL toluene in reaction test tubes. The extraction was done twice and upper toluene layers were collected, combined and evaporated under nitrogen. The dry residue was dissolved in methanol filtered through 0.22 μm membrane and analyzed by HPLC. The HPLC system used was Agilent 1200 equipped with fluorescence detector and autosampler, column Zorbax Eclipse XDB-C18 4.6 × 75 mm 3.5 μm (Agilent Technologies, Santa Clara, CA, USA), mobile phase methanol and water under linear gradient 60% to 95% methanol from 1 to 10 min. Fluorescence detection was conducted at 365 nm excitation wavelength and 510 nm emission wavelength.

### 3.11. Statistical Analysis

The experiment was performed in a completely randomized design. The results presented in the figures are mean values ± standard error based on three replicates. Data were analyzed using two way ANOVA analysis of variance (included in the legends of tables and figures) and Duncan’s multiple range test at *p* ≤ 0.05 with the statistical package STATISTICA 10.0 (Stat-Soft, Inc., Tulsa, OK, USA), marked as footnote letters with the mean values).

## 4. Conclusions

Exposure of seedlings of two wheat cultivars to osmotic stress induced by PEG in hydroponic culture resulted in a greater decrease of gas exchange parameters for resistant CS than for susceptible SQ1 as well as in an increase in water soluble sugars and antioxidant activity in susceptible SQ1 and in proline and ABA content in both cultivars. The present study revealed that exogenous SA and ABA application could counteract the adverse effects of osmotic stress on yield components. The current research findings suggest that they act as osmotic and metabolic regulators and partially as stabilizers of cell components. We also showed that the level of the most important PAs decreased during long-term drought stress applied to wheat. This result confirms that science is still far from providing the complete theory of PA metabolism and their role in the stress response of plants. We also showed that proline is a more universal stress indicator than PAs.

In summary, based on the results of our comprehensive investigations, it can be concluded that SA and ABA ameliorate—mainly in the drought resistant wheat cultivar—the harmful effects and aftereffects of osmotic stress induced by PEG in hydroponics culture through the prevention of ROS accumulation by activating antioxidant system and improving osmotic adjustment.

## Figures and Tables

**Table 1:** Morphological parameters (leaves and roots length) and water content in 28 day old seedlings of two wheat cultivars: SQ1 and CS after seven days of treatment in half-strength Hoagland solution (control, C) and supplemented with PEG, SA (PEG + SA) and ABA (PEG + ABA).

Parameter	Cultivar	Treatment			

		C	PEG	PEG + SA	PEG + ABA
Leaves length (mm)	SQ1	341 ^a,b^ (100%)	258 ^c^ (76%)	276 ^c^ (81%)	252 ^c^ (74%)
	CS	410 ^a^ (100%)	374 ^a^ (91%)	303 ^b,c^ (74%)	341 ^a,b^ (83%)

Roots length (mm)	SQ1	244 ^a,b^ (100%)	152 ^c^ (62%)	202 ^b,c^ (83%)	184 ^b,c^ (75%)
	CS	283 ^a,b^ (100%)	288 ^a^ (102%)	188 ^b,c^ (67%)	221 ^a,b,c^ (78%)

Water content in leaves (%) (WC)	SQ1	85.6 ^a,b^ (100%)	81.0 ^a,b^ (95%)	80.9 ^a,b^ (94%)	78.5 ^b^ (92%)
	CS	87.8 ^a^ (100%)	83.8 ^a,b^ (95%)	82.0 ^c^ (93%)	83.1 ^a,b^ (95%)

Water content in roots [%] (WC)	SQ1	91.9 ^a,b^ (100%)	89.4 ^b,c^ (97%)	85.9 ^c,d^ (93%)	88.1 ^c^ (96%)
	CS	93.0 ^a^ (100%)	89.1 ^a^ (96%)	83.2 ^b,c^ (89%)	87.8 ^c^ (94%)

**Source of variance**	**Leaves length**	**Roots length**	**WC in leaves**		**WC in roots**

Cultivar	***	**	ns		ns
Treatment	***	*	***		***
Cultivar × Treatment	ns	*	**		**

Mean values within lines and cultivars marked with the same letters do not differ signifcantly at *p* ≥ 0.05 according to Duncan’s multiple range test. In parenthesis, percentage values in comparison to the control (C) are given. The results of two way ANOVA analysis of variance are presented in the lower part of the table. The sources of variance for leaves and roots length and for WC in leaves and roots were as follows: two cultivars, four treatments, and interaction between cultivar and treatment.

*, **, *** significant at *p* < 0.05, 0.01, 0.001, respectively; ns, not significant.

**Table 2:** Gas exchange parameters (Pn, E, WUE, g_s_) and chlorophyll content in 28 days old seedlings of two wheat cultivars: CS and SQ1 after seven days of treatment in half-strength Hoagland solution (control, C) and supplemented with PEG, SA (PEG + SA) and ABA (PEG + ABA).

Parameter	Cultivar	Treatment			

		C	PEG	PEG + SA	PEG + ABA
Net photosynthesis (Pn) (μmol CO_2_ cm^−2^ s^−1^)	SQ1	16.20 ^a^ (100%)	11.90 ^d^ (73%)	12.84 ^c,d^ (79%)	11.55 ^d^ (71%)
	CS	17.62 ^a^ (100%)	8.60 ^b,c^ (49%)	9.62 ^b^ (55%)	8.80 ^b,c^ (50%)

Transpiration (E) (mmol H_2_O cm^−2^ s^−1^)	SQ1	5.93 ^a^ (100%)	5.27 ^b^ (89%)	5.50 ^b^ (93%)	4.75 ^b^ (80%)
	CS	7.58 ^b^ (100%)	5.48 ^b^ (72%)	4.84 ^b^ (64%)	4.67 ^b^ (61%)

Water use efficiency (WUE) (μmol CO_2_mmol^−1^ H_2_O)	SQ1	2.78 ^a,b,c^ (100%)	2.45 ^d^ (88%)	2.33 ^c,d^ (84%)	2.54 ^d^ (91%)
	CS	2.35 ^a^ (100%)	1.66 ^a,b^ (71%)	1.99 ^b,c^ (85%)	1.89 ^a,b^ (80%)

Stomatal conductance (g_s_) (mmol H_2_O cm^−2^ s^−1^)	SQ1	117.33^a^ (100%)	65.17 ^c^ (56%)	79.80 ^c^ (68%)	76.83 ^c^ (65%)

	CS	137.83 ^b^ (100%)	64.00 ^c^ (46%)	74.20 ^c^ (54%)	69.67 ^c^ (51%)

Chlorophyll content SPAD	SQ1	6.68 ^a^ (100%)	4.86 ^b,c^ (73%)	5.10 ^b^ (76%)	4.55 ^c^ (68%)

	CS	7.06 ^a^ (100%)	4.84 ^b,c^ (69%)	5,23 ^b,c^ (74%)	4.51 ^b,c^ (64%)

**Source of variance**	**Pn**	**E**	**WUE**	**g_s_**	**SPAD**

Cultivar	***	**	**	***	***
Treatment	**	ns	***	ns	ns
Cultivar × Treatment	*	ns	ns	ns	ns

Mean values within lines and forms marked with the same letters do not differ significantly according at *p* ≤ 0.05 to Duncan’s multiple range test. In parenthesis, percentage values in comparison to the control (C) are given. The results of two way ANOVA analysis of variance are presented in the lower part of the table. The sources of variance for Pn, E, WUE, g_s_ and SPAD were as follows: two cultivars, four treatments, and interaction between cultivar and treatment.

*, **, *** significant at *p* ≤ 0.05, 0.01, 0.001, respectively; ns, not significant.

**Table 3:** Yield components per plant measured in CS and SQ1 cultivars harvested after achieving full maturity in the soil after the replacement from hydroponics culture in four modified media: C, PEG, PEG + SA and PEG + ABA.

Yield component	Cultivar	Treatment			

		C	PEG	PEG + SA	PEG + ABA
Grain number	SQ1	83.3 ^c^ (100%)	49.3 ^c,d^ (59%)	58 ^c,d^ (70%)	42 ^d^ (50%)
	CS	189.3 ^a^ (100%)	135.0 ^b^ (71%)	137.7 ^b^ (73%)	172.7 ^a,b^ (91%)

Grain mass (g)	SQ1	4.0 ^b,c^ (100%)	2.2 ^c,d^ (55%)	2.6 ^c,d^ (65%)	1.9 ^d^ (48%)
	CS	6.6 ^a^ (100%)	4.6 ^a,b^ (70%)	5.0 ^a,b^ (76%)	6.0 ^a^ (91%)

Biomass (g)	SQ1	5.7 ^c,d^ (100%)	3.1 ^d,e^ (54%)	3.7 ^d,e^ (65%)	2.6 ^e^ (46%)
	CS	11.2 ^a^ (100%)	7.6 ^b,c^ (68%)	8.8 ^a,b^ (79%)	10.0 ^a,b^ (89%)

**Source of variance**		**Grain number**	**Grain mass**		**Biomass**

Cultivar		***	***		***
Treatment		ns	*		*
Cultivar × Treatment		ns	ns		ns

Mean values within lines and cultivars marked with the same letters do not differ significantly according at *p* ≤ 0.05 to Duncan’s multiple range test. In parenthesis, percentage values in comparison to the control (C) are given. The results of two way ANOVA analysis of variance are presented in the lower part of the table. The sources of variance for grain and mass number and biomass were as follows: two cultivars, four treatments, and interaction between cultivar and treatment.

*, **, *** significant at *p* ≤ 0.05, 0.01, 0.001, respectively; ns, not significant.
